# Interleukin gene cloning and expression in *E. coli*

**DOI:** 10.1080/07853890.2021.1896889

**Published:** 2021-09-28

**Authors:** Filipa Oliveira, Fernando Cardoso

**Affiliations:** a Faculdade de Ciências e Tecnologia, Universidade Nova de Lisboa (FCT-UNL), Caparica, Portugal; b Instituto de Higiene e Medicina Tropical (IHMT-UNL), UEI Parasitologia Médica, Estrada da Junqueira,100, Lisboa, Portugal; c Global Health and Tropical Medicine (GHTM), Estrada da Junqueira,100, Lisboa, Portugal

## Abstract

**Introduction:**

Cytokines are a large category of protins; they play various functions from inflammation response to cancer development and sepsis. Over-expression or problems in production control of this small peptides can lead to the development of various diseases, including autoimmune diseases, such as rheumatoid arthritis. Inhibition of some of these proteins can result in a therapy that can ease certain symptoms of cases of immunologic diseases.

**Material and Methods:**

This work consists in cloning and expressing six truncated proteins of interleukins: IL-1α, IL-1β, IL-17A, MIF, TNFSF11 and CD20. These synthetic genes were synthesised with the *E. coli* codon usage and inserted in pNZY29 cloning vector (Nzytech). These genes were isolated by amplification by PCR method with specific primers, gel purified and cloned in the pLATE31 (Thermo) expression vector. The purified expression vectors were used to transform the following *E. coli* expression strains: BL21 (DE3), BL21-Gold (DE3), BL21-CondonPlus RIPL (DE3), BL21- Gold (DE3) pLysS, BL21 Star (DE3), BL21 SHuffle, BL21 SHuffle LysY and BL21 XJB (DE3) and expressed and detected according to a previous work [1].

**Results:**

The cloning and expression system used was a directional cloning of PCR-generated fragments and a DNA ligase free method, with this system was achieved a high cloning efficiency (80–90%). The polyacrylamide gel allowed the detection of the strains with higher levels of expression of the recombinant proteins. In this study the level of protein expression was different in each *E. coli* strain tested and was dependent on the protein expressed.

**Discussion and Conclusions:**

We succeeded to produce IL1-α IL1-β IL17A, MIF, TNFSF-11 and CD20 recombinant proteins in different *E. coli* strains but the expression is strain dependent according to the protein. These recombinant proteins are capable of functioning as antigens to produce monoclonal and recombinant antibodies and recombinant peptide ligands. Next steps in the research include culture conditions optimisation to increase recombinant protein yield and the selection and production of recombinant peptide ligands from phage libraries and it is used in an *in vitro* inhibitory and ELISA assay.
